# Eczema Molluscatum in a Patient With Atopic Dermatitis on Upadacitinib and Methotrexate

**DOI:** 10.7759/cureus.115350

**Published:** 2026-08-28

**Authors:** Haley Wiesenhofer, Grant Waldal, Parth Patel, Benjamin Buttars, Carsten R Hamann

**Affiliations:** 1 Dermatology, School of Osteopathic Medicine, Campbell University, Lillington, USA; 2 Dermatology, Arizona State University, Tempe, USA; 3 Dermatology Residency Program, HonorHealth, Scottsdale, USA

**Keywords:** atopic dermatits, eczema molluscatum, jak inhibitor, molluscum contagiosum, rinvoq

## Abstract

Eczema molluscatum refers to widespread molluscum contagiosum (MC) infection occurring in patients with atopic dermatitis (AD), a setting in which impaired skin barrier function and immune dysregulation both promote viral spread. We report the case of a 29-year-old man with longstanding, treatment-refractory AD who was well controlled on upadacitinib, a selective Janus kinase 1 (JAK1) inhibitor, and methotrexate when he developed multiple pink, dome-shaped papules with central umbilication across the cheeks and forehead over a six-month period. The lesions were unresponsive to topical clindamycin-benzoyl peroxide gel. The differential diagnosis included eczema coxsackium, eczema herpeticum, eruptive syringomas, and a refractory JAK inhibitor-associated acneiform eruption, but the absence of pain, systemic symptoms, vesiculation, and follicular morphology supported a diagnosis of eczema molluscatum. The patient was treated with cryotherapy for each lesion, with marked improvement at one-month follow-up. AD itself is a recognized risk factor for MC, reflecting the disease's inherent Th2-skewed, barrier-disrupted immune environment. However, this case raises the possibility that systemic immunosuppression with JAK1 inhibitors, particularly in combination with methotrexate, may further impair type I interferon-mediated antiviral defense and compound susceptibility to MC in patients with AD. Nonetheless, clinicians should maintain a high index of suspicion for eczema molluscatum in patients with AD on JAK inhibitor therapy who develop new facial papules, as these lesions are readily mistaken for a JAK inhibitor-associated acneiform eruption, potentially delaying appropriate treatment.

## Introduction

Molluscum contagiosum (MC) is a viral infection of the skin caused by a poxvirus that presents as clusters of small, round, painless papules with central umbilication [1]. It spreads through skin-to-skin contact, indirect contact with contaminated objects, sexual contact, or autoinoculation via scratching or touching lesions. Although more common in childhood, MC can affect adults, particularly those who are sexually active or immunosuppressed. Eczema molluscatum refers to the occurrence of MC in patients with atopic dermatitis (AD), typically arising in areas of active or previous eczema. The growing use of Janus kinase (JAK) inhibitors for moderate-to-severe AD introduces an additional risk factor for this presentation, as these agents blunt cytokine signaling pathways involved in antiviral immunity. We present a case of diffuse facial eczema molluscatum in a patient with AD receiving upadacitinib and methotrexate, highlighting the diagnostic challenge of distinguishing this eruption from other causes of new facial papules, particularly JAK inhibitor-associated acneiform eruptions.

## Case presentation

A 29-year-old man presented to the dermatology clinic with approximately 20 painless, slightly pruritic papules on the cheeks and forehead that had been present for approximately six months. His medical history was notable for severe AD since childhood, refractory to multiple advanced AD therapies and now well controlled on upadacitinib, a selective JAK1 inhibitor (JAKi), 30 mg daily and methotrexate 15 mg weekly. The patient reported that the papules began approximately six months after starting this regimen. The papules were not responsive to topical clindamycin-benzoyl peroxide (1%/5%) gel.

**Figure 1:**
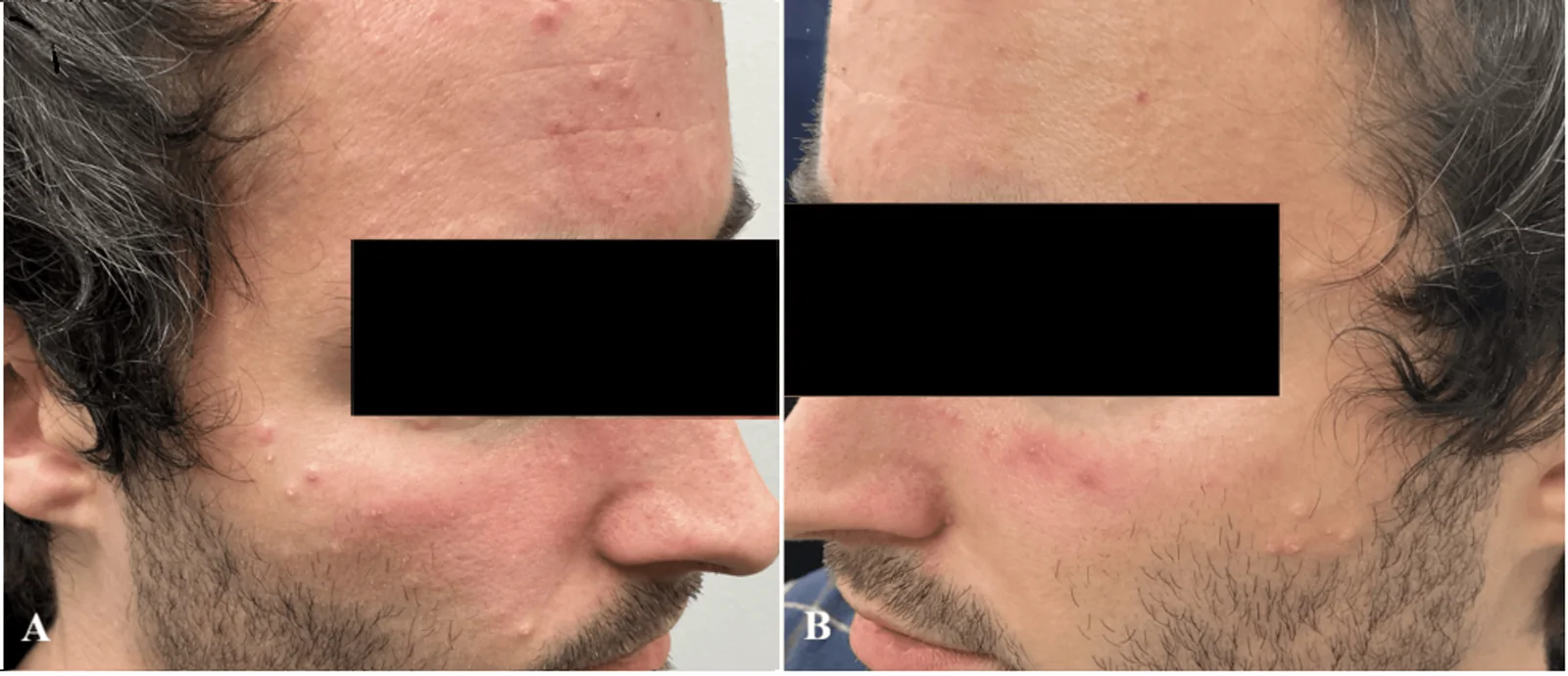
Clinical photographs demonstrating multiple erythematous, umbilicated papules involving the (A) right and (B) left cheek, periorbital regions, and forehead.

Physical examination was notable for pink to skin-colored, dome-shaped papules, some with central umbilication or central accentuation, on the cheeks and forehead (Figure 1A, B). There was no vesiculation, erosion, crusting, or systemic symptoms such as fever or lymphadenopathy. Given this presentation, the differential diagnosis included eczema coxsackium, eczema herpeticum, eruptive syringomas, refractory JAK inhibitor-associated acne, and eczema molluscatum. A diagnosis of eczema molluscatum was made clinically, based on the characteristic umbilicated papular morphology and clinical course of presentation. No dermoscopic, cytologic, or histopathologic testing was performed to confirm the diagnosis.

**Figure 2:**
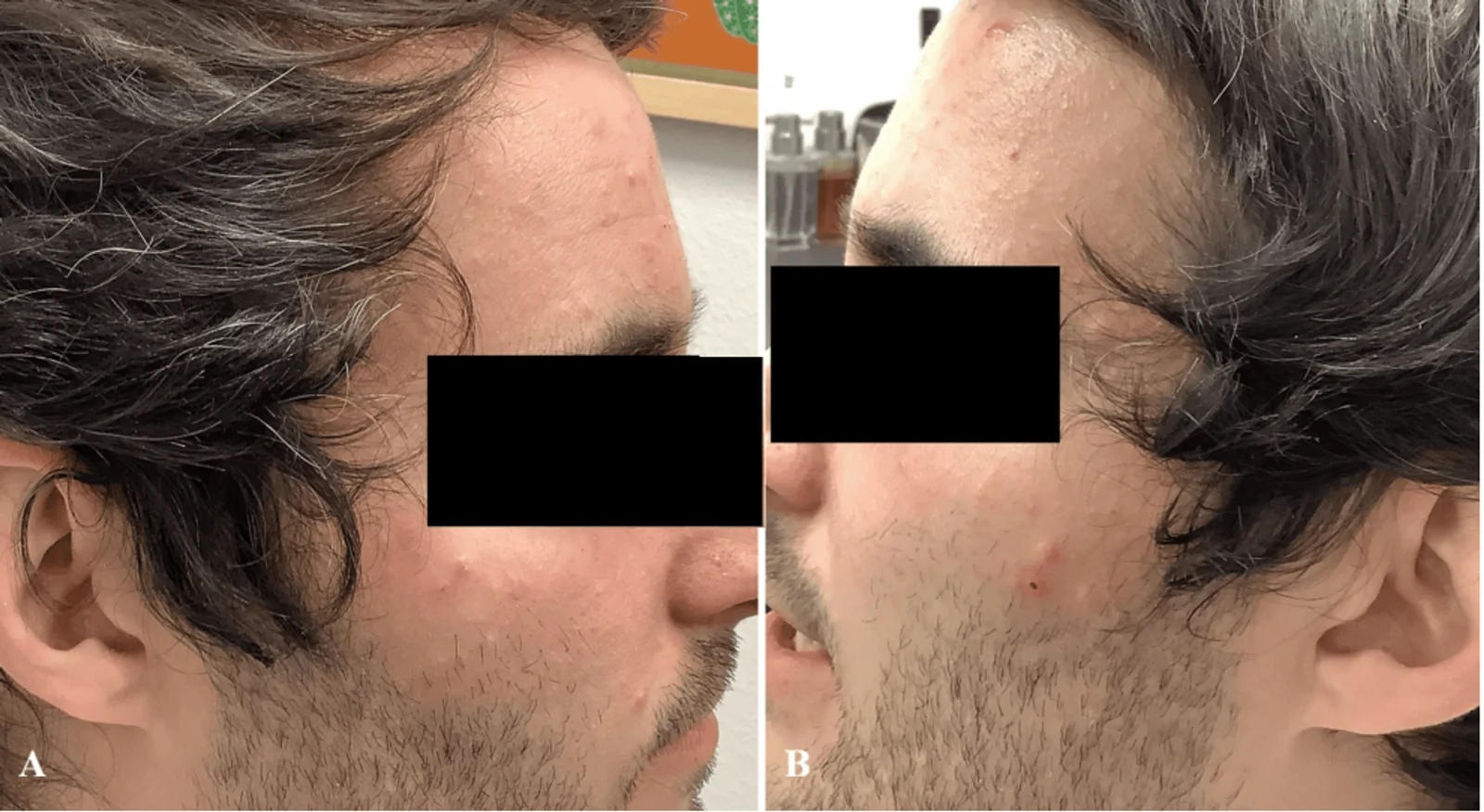
One-month follow-up demonstrating clinical improvement of facial MC lesions following treatment with liquid nitrogen cryotherapy. MC: molluscum contagiosum.

Each lesion was treated with cryotherapy, with significant improvement at one-month follow-up (Figure 2A, B). Residual lesions at follow-up were treated with cryotherapy, and patient was started on tretinoin 0.025% for additional treatment.

## Discussion

Patients with AD are at increased risk of MC due to both skin barrier dysfunction and immune dysregulation. MC virus possesses several mechanisms that allow it to evade the immune system, including inhibition of type I interferon (IFN) signaling [2]. Type I IFNs exhibit antiviral effects by binding to their associated receptors to induce gene expression through the JAK-STAT pathway [3]. Upadacitinib, a selective JAK1 inhibitor, suppresses the JAK-STAT pathway and may further impair type I IFN-mediated responses. In this patient, systemic immunomodulation with upadacitinib in combination with methotrexate likely contributed to diminished antiviral surveillance and increased susceptibility to MC infection. Additionally, because the MC virus enters through epidermal disruption, patients with AD are more predisposed to infection and more widespread disease given their compromised skin barrier [4]. The combination of barrier dysfunction and pharmacologic immunosuppression likely explains the diffuse facial involvement observed in this case.

Differential diagnoses for eczema molluscatum include eczema coxsackium, eczema herpeticum, eruptive syringomas, and, in this setting, refractory JAK inhibitor-associated acne. Eczema coxsackium and eczema herpeticum are cutaneous viral infections caused by coxsackievirus and herpes simplex virus, respectively. Both tend to manifest as monomorphic vesiculopustular or erosive lesions in areas of underlying AD and are often painful rather than pruritic [5,6]. Eczema coxsackium is considered an atypical presentation of hand-foot-and-mouth disease, classically associated with fever, oral ulcers, and vesicular eruptions of the hands, feet, and potentially eczematous areas [5]. Eczema herpeticum, most commonly caused by herpes simplex virus 1, is characterized by punched-out erosions accompanied by systemic symptoms, including fever, lymphadenopathy, and malaise [7]. In our patient, the absence of pain, systemic symptoms, and vesicular or erosive appearance made these diagnoses unlikely.

Eruptive syringomas are rare, benign adnexal neoplasms originating from eccrine sweat ducts that more commonly affect females and present as firm, yellow-brown papules, often in clusters on the neck, chest, or periorbital area [8]. Syringomas typically appear bilaterally in a symmetrical distribution. The central umbilication and distribution of the lesions in our patient were not characteristic of syringomas, making this diagnosis less likely.

Refractory JAK inhibitor-associated acne is one of the most common adverse effects of upadacitinib in patients with AD, presenting as an inflammatory acneiform eruption. Although the pathogenesis remains incompletely understood, JAK inhibition is thought to disrupt pathways involved in epidermal homeostasis, resulting in impaired epidermal repair and altered sebaceous activity [9]. Most cases respond to topical therapies, including benzoyl peroxide, retinoids, and clindamycin. In this case, the morphology of the umbilicated papules and the lack of response to benzoyl peroxide/clindamycin argued against refractory JAK inhibitor-associated acne.

In healthy individuals, MC may resolve spontaneously within several months. However, in immunocompromised patients or those with predisposing risk factors such as AD, lesions tend to be more diffuse, persistent, and treatment resistant [10]. In such cases, treatment is encouraged to reduce possible transmission, limit autoinoculation, and shorten disease duration. Common therapeutic options include cryotherapy, curettage, and topical cantharidin [10,11]. Intralesional Candida injections have also been used in more complicated cases of MC. In this case, each lesion was treated with cryotherapy, with significant improvement at one-month follow-up. Remaining lesions at follow-up were retreated with cryotherapy, and the patient was started on tretinoin 0.025% for additional treatment.

A limitation of this case is that the diagnosis of eczema molluscatum was based on clinical morphology alone; dermoscopic examination and histopathologic confirmation were not performed. While the umbilicated, dome-shaped papular morphology is classically described as diagnostic for MC and was the basis for our clinical diagnosis, dermoscopy can increase diagnostic confidence by revealing features such as central umbilication with surrounding vascular structures, and biopsy remains the definitive standard when the presentation is atypical or the diagnosis is in doubt. In this patient, the absence of vesiculation, systemic symptoms, and follicular involvement made the alternative diagnoses considered above less likely, and the subsequent clinical improvement with cryotherapy was consistent with MC.

## Conclusions

It is important for clinicians to recognize eczema molluscatum in patients with AD on immunosuppressive therapies, especially those on JAK inhibitors, when lesions may be confused with a JAK inhibitor-associated acneiform eruption. Recognizing characteristic lesion morphology, distribution, and clinical evolution is essential to distinguish this infection from treatment-related acneiform eruptions and other clinical mimics, allowing for prompt diagnosis and targeted therapies.
